# Incremental diagnostic value of serum progesterone combined with transvaginal ultrasound in differentiating early ectopic pregnancy: a single-center real-world study

**DOI:** 10.3389/fmed.2026.1949190

**Published:** 2026-10-16

**Authors:** Leilei Liu, Yao Cai, Tingting Wang

**Affiliations:** Department of Ultrasound, Wuhu Fanchang District People’s Hospital, Wuhu, China

**Keywords:** diagnostic value, ectopic pregnancy, M6NP model, progesterone, the location of pregnancy is unknown, transvaginal ultrasound

## Abstract

**Objective:**

To evaluate serum progesterone combined with transvaginal ultrasound for distinguishing ectopic pregnancy (EP) in patients with pregnancy of unknown location (PUL), and to assess diagnostic efficacy across risk stratifications.

**Methods:**

Internally unvalidated analysis included 148 PUL patients (January 2022–December 2025). Final diagnosis by surgical pathology or follow-up divided patients into EP (49) and non-EP (99; intrauterine pregnancy or natural regression). All underwent initial transvaginal ultrasound to confirm PUL. The prediction point was the 48-hour reassessment. Predictors were abnormal adnexal findings on follow-up ultrasound at 48 h, serum progesterone, and 48-hour *β*-hCG results available at that visit. The intended application is risk re-stratification and follow-up planning at 48-hour reassessment, not replacement of surgical/histopathological diagnosis or immediate treatment. Progesterone and *β*-hCG were measured; groups were compared. Logistic regression screened independent predictors. ROC curves evaluated single and combined indicators. We assessed if progesterone improved the ultrasound + M6NP model, with M6NP defined as M6 excluding initial progesterone.

**Results:**

The EP group had lower serum progesterone, initial *β*-hCG, 48-hour *β*-hCG change rate, and endometrial thickness, and higher abnormal adnexal ultrasound detection (*P* < 0.05). Multivariate analysis showed log progesterone, abnormal adnexal ultrasound, previous EP, and M6NP high-risk classification (≥5%) were independent predictors (*P* < 0.05). All AUC values are apparent/in-sample estimates. Ultrasound + M6NP had an apparent AUC of 0.913; adding progesterone increased it to 0.947. In the M6NP high-risk subgroup, progesterone plus ultrasound had an apparent AUC of 0.945 and specificity of 91.67%. Because the model was developed and evaluated in the same dataset without bootstrap or internal validation, estimates are preliminary and require external validation.

**Conclusion:**

Internally unvalidated analysis, serum progesterone combined with transvaginal ultrasound was associated with improved apparent diagnostic accuracy for EP in PUL, especially within M6NP risk stratification. These estimates are apparent/in-sample, preliminary, and hypothesis-generating.

## 1 Introduction

Ectopic pregnancy (EP) refers to the implantation of a fertilized egg outside the uterine cavity, with tubal pregnancy being the most common, accounting for over 95% of all EP cases (1, 2). EP is one of the major causes of early pregnancy mortality, and early diagnosis and intervention are crucial for reducing maternal mortality and preserving fertility (3). In recent years, with the widespread application of assisted reproductive technology (ART), the incidence rate of EP is on the rise. It has been reported that the incidence of EP in the ART cycle can reach 2%–5%, significantly higher than that of natural pregnant people (3). However, the early symptoms of EP are often atypical, and many patients present with a pregnancy of unknown location (PUL) as the initial manifestation, posing a challenge for clinical differentiation (4).

At present, the main methods for distinguishing EP from normal intrauterine pregnancy include dynamic monitoring of serum *β* - human chorionic gonadotropin (*β* - hCG), measurement of serum progesterone, and transvaginal ultrasound examination (5). The 48 h change rate of *β* - hCG has been widely used to distinguish between viable and non viable pregnancies, but its false positive rate cannot be ignored. Serum progesterone, as a direct reflection of luteal function, is usually at a lower level during EP, which has become widely recognized (6). However, there are significant individual differences in progesterone levels, and the diagnostic efficacy of a single measurement is limited. Transvaginal ultrasound can directly observe abnormal structures in the adnexa region and endometrial thickness, but the sonographic features of early EP are not always clear and distinguishable (7). When various diagnostic methods are used alone, their sensitivity and specificity are difficult to meet clinical needs, and their specific contribution weights need to be further clarified, which is a clinically relevant limitation.

In recent years, the introduction of predictive models has provided new ideas for risk stratification of PUL. In recent years, the introduction of predictive models has provided new ideas for risk stratification of PUL. The M6NP model (the M6 model excluding initial progesterone) is an hCG-based model derived from the M6 framework (8). The M6NP model uses the log-transformed initial *β*-hCG concentration together with the log-transformed 48-hour/initial *β*-hCG ratio, i.e., log(hCG ratio), and its quadratic term, exactly as specified in Section 2.4. The original M6 model was developed by Van Calster et al. (8) using the log-transformed initial *β*-hCG concentration. Age, vaginal bleeding, and previous ectopic pregnancy were not included in the original M6 model; these clinical factors were evaluated in the subsequent model update by Kyriacou et al. (9). The M6NP variant excludes initial progesterone from the model. This model has been externally validated in several PUL cohorts (7, 10). The promotion of this model helps to reduce unnecessary follow-up and intervention. However, even with the help of risk stratification, some EPs are still misdiagnosed as low-risk pregnancies. How to further improve diagnostic accuracy on the basis of the existing risk stratification system has become a key issue that urgently needs to be addressed (10). A small number of studies have attempted to combine progesterone, ultrasound features, and predictive models, but their incremental diagnostic value has not been fully validated (11). Especially in the Chinese population, the systematic evaluation of the combined diagnosis strategy of progesterone and ultrasound is still very limited, and the source of this heterogeneity may play a key role in the differences in subject characteristics (12).

The limitations of existing research are mainly reflected in the following aspects. Firstly, most studies are retrospective analyses with small sample sizes, lacking support from rigorous efficacy calculations, and the robustness of statistical conclusions is questionable (13). Secondly, the research population is mostly Caucasian or African American, and diagnostic thresholds and model validation data for Asian populations, especially Chinese patients, are extremely scarce. In addition, the progesterone detection methods and cutoff values used in different studies vary, making it difficult to directly compare and generalize the results. Some reports tend to support the effect of progesterone alone in diagnosis, but there are also attempts that have not observed positive results (14). More importantly, there is a lack of incremental value research that organically combines progesterone, ultrasound features, and modern predictive models (such as M6NP), which cannot clearly answer the core question of “how much clinical benefit can be brought by adding progesterone”.

The aim of this study is to evaluate the incremental diagnostic value of serum progesterone combined with transvaginal ultrasound in distinguishing early ectopic pregnancy, and explore its application efficacy under the risk stratification framework of M6NP model. The study adopted a retrospective design from the real world and included 148 patients with unknown or suspected pregnancy location EP. By constructing a multi parameter combination model that includes progesterone and ultrasound, the diagnostic advantages of this model over traditional single indicators were quantified. We hope that the results of this study can provide evidence-based diagnosis for early EP in the Chinese population, and also provide new ideas for optimizing clinical decision-making pathways.

## 2 Data and methods

### 2.1 General information

This study is a single center, retrospective, real-world research. The research plan has been approved by the Medical Ethics Committee of Wuhu Fanchang District People's Hospital (approval number: 2026LL07003) and follows the latest revised version of the Helsinki Declaration. Due to the retrospective nature of the study, the requirement for patients to sign an informed consent form has been waived by the Medical Ethics Committee because the study involved no more than minimal risk to participants and the waiver did not adversely affect the rights and welfare of the participants. The research period is from January 2022 to December 2025.

Sample size justification: This retrospective sample-size calculation was based on the originally planned comparison of serum progesterone levels between ectopic pregnancy (EP) and non-EP groups, not on the primary incremental diagnostic model analysis. Based on previously reported data, the median serum progesterone level in patients diagnosed with EP is approximately 3.81 nmol/L, whereas the median level in the non-EP group is approximately 17.70 nmol/L (15). The Cohen's d of 0.6 was approximated from the ratio of the difference in medians to the pooled standard deviation reported in the cited study; however, because the source values were medians rather than means, this effect size is approximate. Assuming an EP to non-EP ratio of 1:2, 80% power, and a two-sided *α* of 0.05, the required sample size was at least 129 participants. The total of 148 included patients was a retrospective convenience sample and was not prospectively powered for multivariable model development, incremental AUC testing, or subgroup analyses. The number of outcome events relative to the number of candidate predictors was limited, and therefore all multivariable performance estimates should be considered preliminary.

Patient screening and grouping: Female patients who received serum *β* - human chorionic gonadotropin (*β* - hCG) and progesterone testing for “unknown pregnancy location” or “suspected ectopic pregnancy” at our gynecology emergency or outpatient department from January 2022 to December 2025, and underwent transvaginal ultrasound examination during the same period, were included. According to the final confirmed pregnancy outcome of the patients, they were divided into two groups: ectopic pregnancy group (EP group, *n* = 49 cases) and non ectopic pregnancy group (non EP group, *n* = 99 cases, including intrauterine pregnancy and naturally receding pregnancy with unknown location). The specific process is shown in Figure 1.

**Figure 1:**
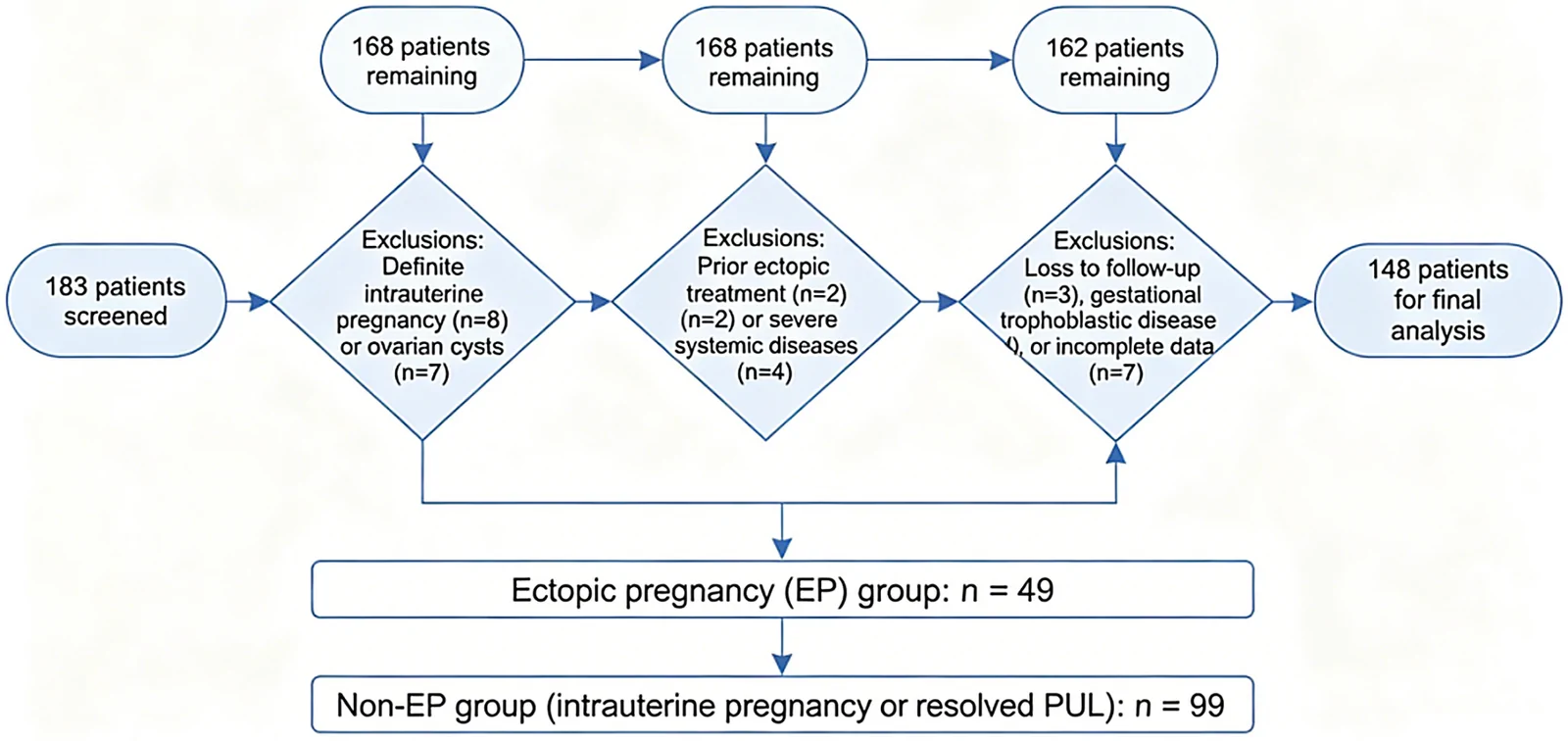
The specific process.

### 2.2 Inclusion and exclusion criteria

Inclusion criteria:

(1) Age range: 18–45 years old, with a history of sexual activity, and a positive result in *β* - hCG test (>5 IU/L);
(2) At the initial transvaginal ultrasound examination, patients met the criteria for a pregnancy of unknown location (PUL), defined as a positive serum *β*-hCG test with no evidence of an intrauterine gestational sac and no adnexal mass or other direct sonographic sign suggestive of an ectopic pregnancy. Abnormal adnexal ultrasound findings, used as a predictor in this study, were defined as suspicious adnexal features observed on the follow-up transvaginal ultrasound performed at the 48-hour reassessment visit (e.g., heterogeneous adnexal mass, tubal ring sign, or 'doughnut' sign). Thus, the initial PUL definition and the subsequent abnormal adnexal findings are not contradictory; they reflect the evolving sonographic appearance of an early ectopic pregnancy;
(3) We have completed the complete diagnostic evaluation process in our hospital, including initial serum *β* - hCG and progesterone level measurements, as well as serum *β* - hCG follow-up after 48 h, and based on this, the final pregnancy outcome is determined;
(4) All clinical data, laboratory test results, and imaging images are complete and traceable.

Exclusion criteria:

(1) Diagnosed with intrauterine pregnancy and clear fetal heartbeat visible on ultrasound;
(2) Diagnosed with gestational trophoblastic tumors such as complete molar pregnancy, invasive molar pregnancy, choriocarcinoma, etc;
(3) Received any medication (such as methotrexate) or surgical treatment for ectopic pregnancy before enrollment;
(4) There are clear functional ovarian cysts or other adnexal space occupying lesions that may affect ultrasound diagnosis;
(5) Individuals with severe liver and kidney dysfunction, hematological disorders, or malignant tumors;
(6) Due to incomplete data (such as missing beta hCG data at key time points or unclear ultrasound images), it is impossible to determine the final pregnancy outcome.

### 2.3 Research methods

All patients undergo the following standardized assessment process at their first visit:

(1) Serological testing: On the day of treatment, elbow vein blood was collected and electrochemiluminescence immunoassay (Roche Cobas e601 fully automatic electrochemiluminescence immunoassay analyzer, equipped with Roche original reagents) was used to detect serum levels of *β* - human chorionic gonadotropin and progesterone. The testing is strictly operated according to the instructions, and the coefficient of variation within and between batches is less than 5%. Progesterone is expressed in nanomoles per liter (nmol/L).
(2) Imaging evaluation: Two ultrasound physicians with more than 5 years of experience in obstetrics and gynecology used the same Philips EPIQ 5 color Doppler ultrasound diagnostic instrument (RIC5–9-D intracavitary probe, frequency 5–9 MHz) to independently perform transvaginal ultrasound examination without knowing the patient's laboratory test results. Any disagreement between the two operators regarding categorical ultrasound findings was resolved by consensus after joint review of the stored images. Interobserver agreement was not formally assessed in this retrospective study; therefore, Cohen's kappa is not reported. In addition to the initial scan used to confirm PUL, a follow-up transvaginal ultrasound examination was performed at the 48-hour reassessment visit. The prediction time point for the combined diagnostic model was completely and explicitly defined as the 48-hour reassessment visit. At this prediction time point, the abnormal adnexal ultrasound findings used in the model were those obtained on the follow-up transvaginal ultrasound performed at the 48-hour reassessment, and they were used together with serum progesterone and the 48-hour serum *β*-hCG results available at the same visit. All predictor variables, including abnormal adnexal ultrasound findings, were ascertained at this 48-hour prediction time point and before any clinical decision regarding surgical intervention. The abnormal adnexal ultrasound finding used in the analysis was defined as any suspicious adnexal finding observed on this 48-hour follow-up scan, not on the initial PUL-confirming scan. The intended clinical application is therefore to provide risk re-stratification and follow-up planning for women with PUL at the 48-hour reassessment visit, after the 48-hour *β*-hCG result and the follow-up transvaginal ultrasound are available; the model is not intended for use at the initial visit, is not a stand-alone diagnostic test, and does not replace surgical or histopathological confirmation or clinical judgment. Observe and record the following indicators: endometrial thickness (EMT), endometrial morphology (three-line sign/non three-line sign), presence of gestational sac (or false gestational sac) in the uterus, presence of lumps in the bilateral adnexa (and their morphological characteristics, such as "tubal ring sign", "doughnut sign", etc.), and presence of free fluid in the abdominal cavity. The thickness of the endometrium is measured on the sagittal plane at the thickest point of the bilayer endometrium.
(3) Pregnancy outcome determination: The final diagnosis was established by a senior gynecologist blinded to the initial evaluation, using all available clinical data except the M6NP model prediction. The reference standard for ectopic pregnancy was confirmation of an extrauterine gestational sac by laparoscopy or laparotomy with histopathological evidence. All 49 ectopic pregnancy cases in this study were confirmed surgically and histopathologically; no EP cases were classified solely by clinical follow-up. For patients managed expectantly, the final outcome was determined by serial serum *β*-hCG measurements together with transvaginal ultrasound examinations until the pregnancy location was definitively visualized, the *β*-hCG level declined to undetectable levels, or a viable intrauterine pregnancy was confirmed, in accordance with the National Institute for Health and Care Excellence (NICE) diagnostic algorithms for pregnancy of unknown location. In the non-EP group, intrauterine pregnancy was defined as sonographic confirmation of an intrauterine gestational sac with or without fetal cardiac activity. Spontaneously resolving pregnancy of unknown location was defined as serial *β*-hCG levels declining to undetectable levels without sonographic evidence of intrauterine or extrauterine pregnancy at any follow-up assessment. Because a spontaneously resolving PUL may occasionally represent a spontaneously resolving ectopic pregnancy, this classification may be subject to misclassification bias, which is addressed in the Limitations section. The M6NP model was not used for final outcome adjudication.

### 2.4 Observation indicators

This study included the following 10 observation indicators:

(1) Serum progesterone level: measured at the first visit (nmol/L). Numerous studies have shown that progesterone levels in EP patients are significantly lower than those in normal intrauterine pregnancies.
(2) Initial serum *β* - human chorionic gonadotropin level: measured at first visit (IU/L). EP patients often exhibit low or slow increase in *β* - hCG levels.
(3) 48 h serum *β* - human chorionic gonadotropin level: serum *β* - hCG level (IU/L) rechecked after 48 h. Used for calculating dynamic changes.
(4) Dynamic changes of *β* - human chorionic gonadotropin: i.e., (*β* - hCG48- *β* - hCG0)/*β* - hCG0×100%, used to evaluate its trend over time. A normal intrauterine pregnancy typically results in an increase of ≥ 53% in *β* - hCG within 48 h, while EP increases slowly or decreases.
(5) Endometrial thickness: The thickness of the endometrium in the longitudinal section of the uterus was measured by transvaginal ultrasound (mm). The endometrial thickness of EP patients is usually thinner, with studies showing an EMT of 8.3 ± 3.37 mm in the EP group, significantly lower than the 16.12 ± 3.09 mm in the normal pregnancy group.
(6) Abnormal ultrasound features in the adnexal region: defined as the sonographic characteristics of any suspected ectopic pregnancy observed through transvaginal ultrasound at the 48-hour reassessment visit, such as heterogeneous masses, vacuolar signs, tubal ring signs, etc. in the adnexal region. Record as present/absent.
(7) Progesterone/*β* - human chorionic gonadotropin ratio: Calculate the ratio of serum progesterone to *β* - hCG at the first visit (nmol/L vs IU/L) as a potential co diagnostic indicator. Some studies suggest that the P/*β* - hCG ratio helps distinguish between EP and intrauterine pregnancy.
(8) The M6NP predicted probability: The predicted probability of ectopic pregnancy was calculated for each patient using the original published M6NP multinomial logistic regression model, which is an hCG-based model derived from the M6 framework (the M6 model excluding initial progesterone) (8). The hCG ratio was defined as the serum *β*-hCG level at 48 h divided by the initial serum *β*-hCG level. In the published M6NP model, EP is the reference outcome, and two multinomial linear predictors are used, for FPUL versus EP and IUP versus EP, respectively. The exact published M6NP equations are as follows:

z₁ = log(P(FPUL)/P(EP)) = 2.5506−0.4242   ×   log(initial hCG)−2.9502 × log(hCG ratio) + 2.1765 × [log(hCG ratio)]2

z₂ = log(P(IUP)/P(EP)) = −3.2842 + 0.4072 × log(initial hCG) + 1.9238 × log(hCG ratio) + 2.8952 × [log(hCG ratio)]2

where log refers to the natural logarithm. The predicted probabilities for each outcome were obtained as:

P(FPUL) = exp(z₁)/[1 + exp(z₁) + exp(z₂)]

P(IUP) = exp(z₂)/[1 + exp(z₁) + exp(z₂)]

P(EP) = 1/[1 + exp(z₁) + exp(z₂)]

The numerical coefficients were those published in the original M6 development study by Van Calster et al. (2016) (8), and the M6NP model formula was independently validated based on the equation published by Fistouris et al. We confirm that the M6NP predicted probabilities used in the present analysis were calculated using these exact published equations. Calculations were performed in IBM SPSS 26.0 and independently checked in Microsoft Excel using the spreadsheet equations specified above; no separate commercial M6NP software package was used. The M6NP model does not include age, vaginal bleeding, or previous ectopic pregnancy as predictors; these clinical factors were evaluated in the subsequent model update by Kyriacou et al. (2024) (9) and were not used in the present study. For univariate and receiver operating characteristic analyses, the M6NP continuous predicted probability was used. For the multivariable logistic regression model, the published M6NP threshold of ≥5% was applied to define a binary high-risk variable, consistent with the prespecified risk-stratification framework.

Outcome variable:

(9) Primary endpoint: Diagnostic performance for distinguishing ectopic pregnancy (yes/no), including sensitivity, specificity, positive predictive value, and negative predictive value.
(10) Secondary endpoint: Compare the differences in ultrasound and serological indicators among the groups at the initial visit; Evaluate the incremental diagnostic value of progesterone combined with ultrasound indicators compared to using one alone (quantified by the area under receiver operating characteristic (ROC) curve and the integrated discrimination improvement (IDI)).

### 2.5 Statistical methods

All data were analyzed using IBM SPSS 26.0 and MedCalc 20.0 statistical software. Quantitative data that conforms to normal distribution are expressed as mean ± standard deviation, and independent sample t-test is used for inter group comparison; Non normally distributed quantitative data are represented by median (interquartile range) [M (P25, P75)], and Mann Whitney U test is used for inter group comparison. Count data is presented in terms of examples (percentages), and comparison between groups is conducted using the chi square test or Fisher's exact probability method.

For the primary analysis, we prespecified a clinically driven logistic regression model based on prior evidence and biological plausibility. The candidate predictors were history of ectopic pregnancy, history of pelvic inflammatory disease, log-transformed serum progesterone, endometrial thickness, abnormal adnexal ultrasound findings, and the M6NP high-risk classification (≥5%). Because initial *β*-hCG, 48-hour *β*-hCG, and *β*-hCG change rate were highly correlated with the M6NP predicted probability, only the M6NP high-risk classification was retained in the multivariable model to avoid multicollinearity, while the continuous M6NP predicted probability was used in univariate and ROC analyses to preserve information. History of pelvic inflammatory disease and gravidity were not forced into the final model because they were strongly associated with abnormal adnexal ultrasound findings or were considered surrogate markers for prior pregnancy-related risk and did not materially improve model discrimination in this dataset. Before model construction, multicollinearity among candidate predictors was assessed using variance inflation factors (VIF). Because initial *β*-hCG, 48 h *β*-hCG, and *β*-hCG change rate were highly correlated with the M6NP high-risk classification, only the most clinically integrative variable (M6NP high-risk classification) was retained to avoid multicollinearity (VIF >5). The M6NP high-risk classification (≥5%) was entered as a binary variable in the multivariable model to align with clinical risk stratification and to improve model stability given the limited number of events. All model predictions and combined diagnostic strategies in this study are anchored at the 48-hour reassessment visit, and the intended clinical application is to support risk re-stratification and follow-up planning for women with PUL at that time point rather than to guide immediate treatment at the initial visit or to replace definitive diagnostic confirmation. The M6NP continuous predicted probability was retained in univariate analyses and ROC curve construction to preserve information. Continuous variables were entered as original values, except for serum progesterone, which was log-transformed (Log progesterone) to improve the linearity of its relationship with the log odds of ectopic pregnancy because the raw distribution was markedly right-skewed. Linearity in the logit for continuous predictors was assessed using restricted cubic splines; no evidence of a non-linear relationship was found for log progesterone or the M6NP continuous predicted probability in this dataset. Categorical variables were coded as dummy variables. The odds ratio (OR) and its 95% confidence interval (CI) were calculated. Using receiver operating characteristic (ROC) curve analysis to evaluate the diagnostic efficacy of each independent indicator and its combined indicators, calculate the area under the curve (AUC), sensitivity, specificity, positive predictive value, and negative predictive value at the optimal cutoff value. The optimal cutoff value for the combined models was determined by maximizing the Youden index in the same dataset; therefore, sensitivity and specificity at these cutoffs are subject to optimism and should be interpreted with caution. Because of the limited number of events, all diagnostic performance estimates, should be interpreted as exploratory. 95% confidence intervals for AUC were derived from ROC analysis, and 95% confidence intervals for sensitivity, specificity, positive predictive value, and negative predictive value for all diagnostic indicators were calculated using the Wilson score method. These intervals were not reported for the small subgroup analyses because they were considered unstable in those subgroups. For the main model comparison (Model B: ultrasound + M6NP + progesterone), the Wilson score method was also used for sensitivity, specificity, PPV, and NPV, and the intervals were verified against the Wilson formula. Evaluate the incremental diagnostic value of progesterone by comparing the area under the ROC curves constructed based on logistic regression models with and without progesterone. All hypothesis tests are two-sided tests, with *P* < 0.05 indicating statistically significant differences.

## 3 Results

### 3.1 Baseline data comparison

Compare the baseline data of 148 patients included in this study. The patients in the ectopic pregnancy (EP) group and the non ectopic pregnancy (non EP) group were balanced and comparable in terms of age, body mass index, number of pregnancies, and duration of amenorrhea, with no statistically significant differences (*P* > 0.05). Of the 99 non-EP patients, 62 had an intrauterine pregnancy (62.6%) and 37 had a spontaneously resolving pregnancy of unknown location (37.4%). In terms of clinical characteristics, the serum progesterone, initial and 48 h levels of *β* - human chorionic gonadotropin (hCG), 48 h increase in *β* - hCG, and endometrial thickness in the EP group were significantly lower than those in the non EP group; At the same time, the proportion of patients with a history of ectopic pregnancy, pelvic inflammatory disease, abnormal ultrasound signs in the adnexa region, and M6NP model assessment as high-risk in the EP group was significantly higher than that in the non EP group (*P* < 0.05). See Table 1 and Figures 2, 3.

**Table 1:** Comparison of baseline characteristics between two groups of patients.

Variable	EP Group (*n*=49)	Non-EP Group (*n*=99)	Statistic	*P* value
Age (years), Mean ± SD	29.50 ± 5.80	28.94 ± 5.60	t = 0.566	0.572
BMI (kg/m^2^), Mean ± SD	22.80 ± 3.20	23.00 ± 3.42	t = −0.342	0.733
Gravidity, Median (Q1, Q3)*	2 (1, 4)	2 (1, 3)	Z = −1.912	0.056
Prior ectopic pregnancy, *n* (%)	12 (24.49)	8 (8.08)	*χ*^2^ = 7.549	0.006
History of PID, *n* (%)	18 (36.73)	13 (13.13)	χ^2^ = 11.028	<0.001
Days of amenorrhea, Median (Q1, Q3)*	44 (38, 51)	43 (39, 48)	Z = −0.585	0.559
Serum progesterone (nmol/L), Median (Q1, Q3)*	10.80 (7.50, 15.20)	48.50 (32.00, 65.80)	Z = −9.032	<0.001
Initial *β*-hCG (IU/L), Median (Q1, Q3)*	780 (420, 1,450)	1,850 (950, 3,950)	Z = −4.717	<0.001
48-hour β-hCG (IU/L), Median (Q1, Q3)*	890 (460, 1,650)	2,820 (1,360, 5,880)	Z = −5.956	<0.001
β-hCG change rate (%), Mean ± SD	15.20 ± 22.50	58.30 ± 30.10	t = −8.866	<0.001
Endometrial thickness (mm), Mean ± SD	8.30 ± 3.37	16.12 ± 3.09	t = −14.057	<0.001
Abnormal adnexal ultrasound, *n* (%)	39 (79.59)	9 (9.09)	χ^2^ = 74.334	<0.001
P/β-hCG ratio, Median (Q1, Q3)*	0.014 (0.010, 0.019)	0.028 (0.020, 0.044)	Z = −1.479	0.139
M6NP high-risk, *n* (%)	41 (83.67)	12 (12.12)	χ^2^ = 73.001	<0.001

*Representing skewed distribution, expressed as median (interquartile range), Mann–Whitney U test is used for inter group comparison. Z-value is approximate.

**Figure 2:**
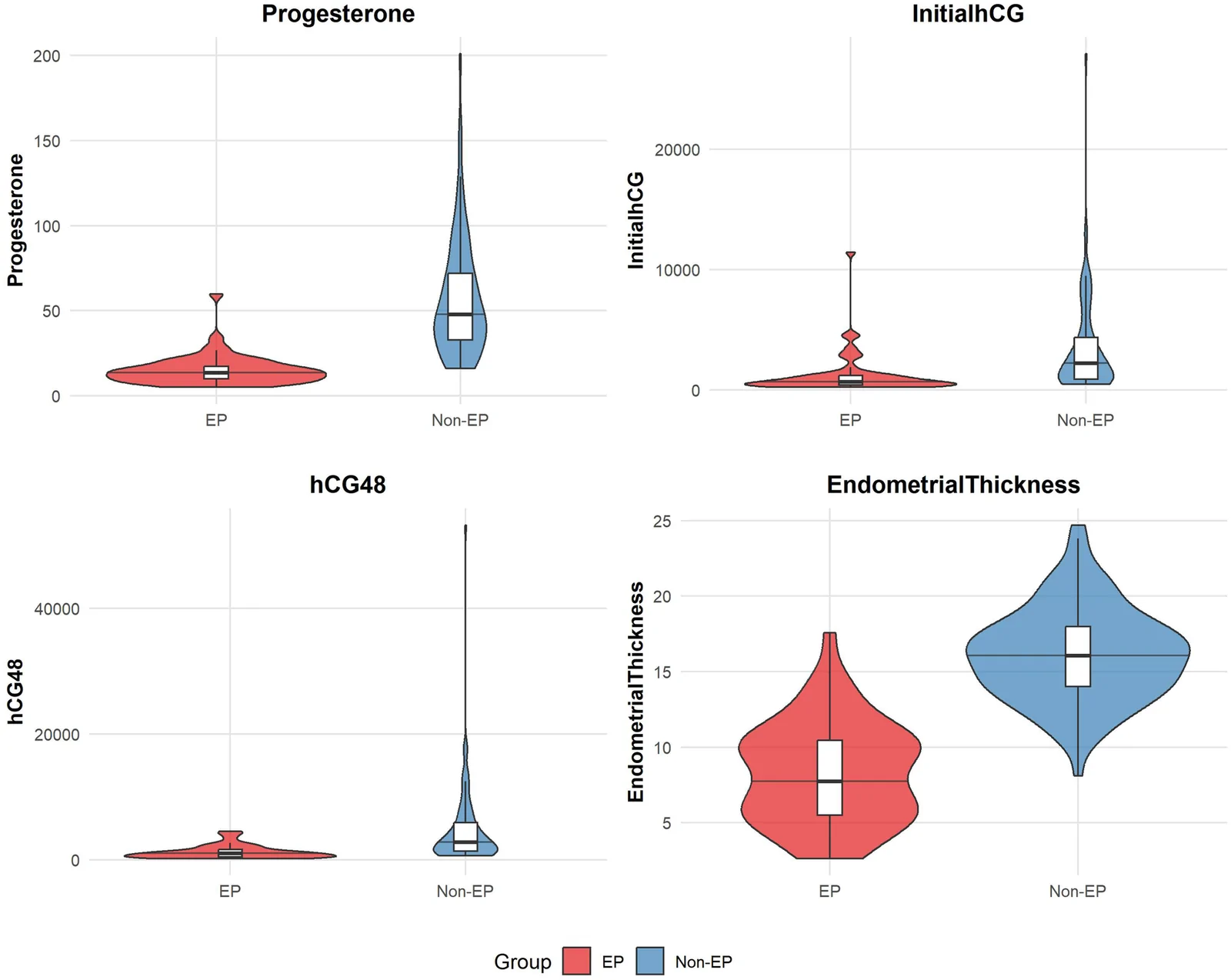
Comparison of continuous clinical and laboratory variables between the EP and non-EP groups.

**Figure 3:**
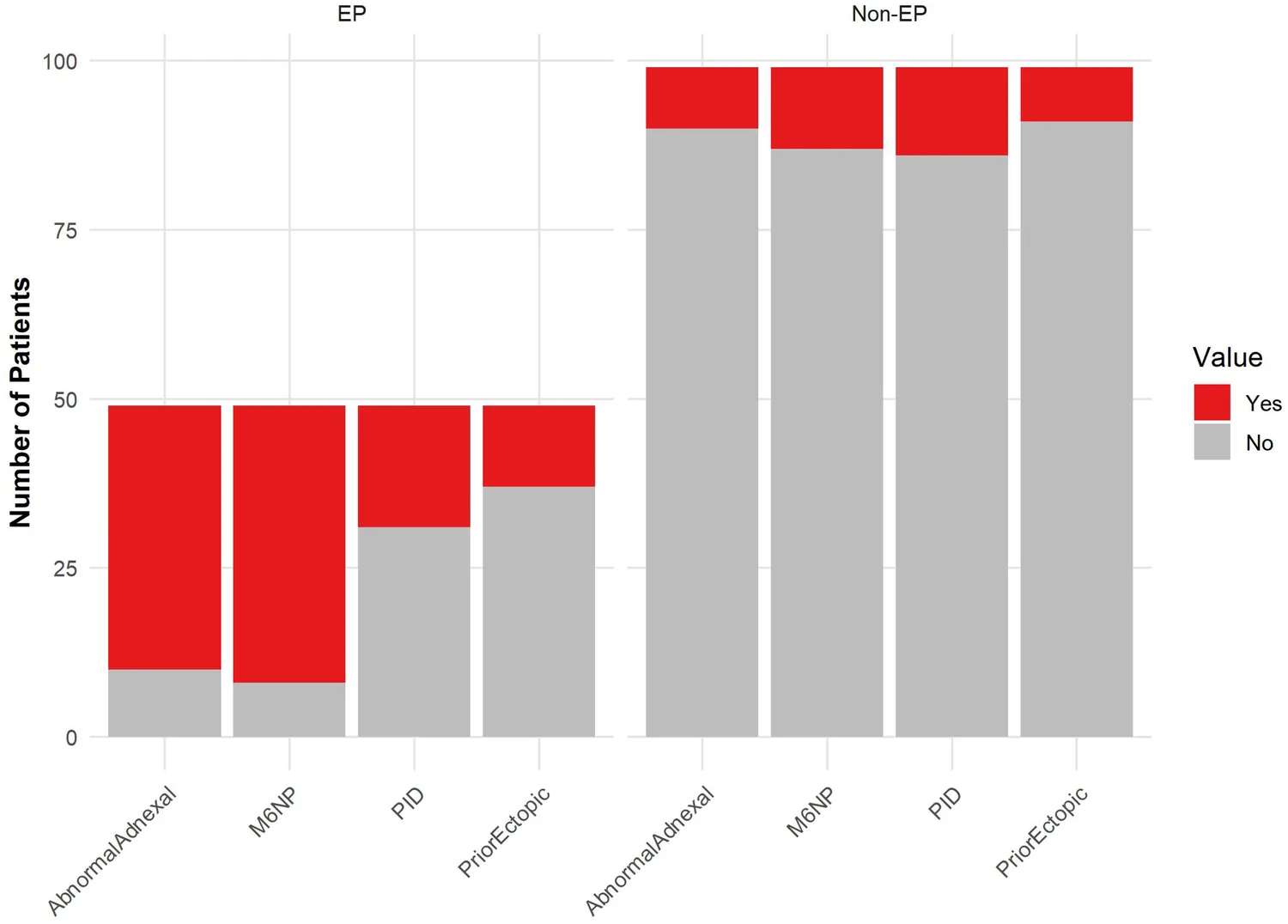
Comparison of categorical clinical characteristics between the EP and non-EP groups.

### 3.2 Univariate logistic regression analysis

To screen and identify potential predictive factors for EP, perform univariate logistic regression analysis on all variables in Table 1. The results showed (Table 2) that a history of ectopic pregnancy, pelvic inflammatory disease, serum progesterone (logarithmic conversion), initial *β* - hCG (logarithmic conversion), 48 h *β* - hCG (logarithmic conversion), dynamic changes in *β* - hCG, endometrial thickness, abnormal ultrasound features in the adnexal region, and the M6NP continuous predicted probability were all significant predictive factors for EP (*P* < 0.05). The OR values for age, body mass index, duration of amenorrhea, and P/*β*-hCG ratio were not significant.

**Table 2:** Prediction factors for identifying ectopic pregnancy using univariate logistic regression analysis.

Variable	β	SE	Wald χ^2^	OR (95% CI)	*P* value
Age	0.018	0.032	0.32	1.018 (0.956–1.084)	0.572
Body mass index	−0.019	0.055	0.12	0.981 (0.881–1.093)	0.73
Gravidity	0.256	0.134	3.65	1.292 (0.993–1.681)	0.056
Prior ectopic pregnancy	1.305	0.496	6.91	3.689 (1.395–9.755)	0.008
History of PID	1.346	0.42	10.26	3.841 (1.686–8.749)	0.001
Log progesterone	−1.874	0.342	30.02	0.154 (0.079–0.301)	<0.001
Log initial β-hCG	−0.831	0.198	17.61	0.436 (0.296–0.642)	<0.001
Log 48hour β-hCG	−1.027	0.217	22.39	0.358 (0.234–0.548)	<0.001
β-hCG dynamic change	−0.038	0.008	23.84	0.963 (0.948–0.977)	<0.001
Endometrial thickness	−0.298	0.065	21.32	0.742 (0.653–0.843)	<0.001
Abnormal adnexal ultrasound	3.664	0.498	54.14	39.00 (14.70–103.50)	<0.001
P/β-hCG ratio	0.012	0.008	2.18	1.012 (0.996–1.028)	0.14
M6NP predicted probability (per 0.10-unit increase)	1.267	0.189	44.90	3.55 (2.46–5.12)	<0.001

### 3.3 Multivariate logistic regression analysis

Table 3 shows the results of the multivariate logistic regression analysis, with the occurrence of ectopic pregnancy as the dependent variable (assigned values: 0 = intrauterine pregnancy, 1 = ectopic pregnancy). Variables were selected for the multivariable model based on a prespecified, clinically driven strategy informed by prior evidence and biological plausibility, as detailed in the Statistical Methods. The candidate predictors were history of ectopic pregnancy, history of pelvic inflammatory disease, log-transformed serum progesterone, endometrial thickness, abnormal adnexal ultrasound findings, and the M6NP high-risk classification (≥5%). Because of high collinearity between *β*-hCG parameters (initial *β*-hCG, 48 h *β*-hCG, *β*-hCG change rate) and the M6NP high-risk classification, the individual *β*-hCG variables were not forced into the multivariable model; instead, the M6NP high-risk variable was retained as a composite predictor. Continuous variables were entered as original values except for progesterone, which was log-transformed, and categorical variables were coded as dummy variables (0 = no, 1 = yes). The results showed that a history of ectopic pregnancy, logarithmic conversion value of progesterone, abnormal ultrasound signs in the adnexa region, and the M6NP high-risk classification were independent predictive factors for ectopic pregnancy (*P* < 0.05). Among them, a history of ectopic pregnancy, abnormal ultrasound signs in the adnexa region, and the M6NP high-risk classification (≥5%) were independent risk factors for ectopic pregnancy, while increasing progesterone levels were inversely associated with the odds of ectopic pregnancy; the association between endometrial thickness and ectopic pregnancy was not statistically significant (*P* > 0.05). See Table 3 and Figure 4. Figure 4 shows the apparent/in-sample calibration plot for the final combined model (progesterone + ultrasound + M6NP). Because this is an apparent calibration plot, the calibration intercept and slope are 0.00 and 1.00 by definition. Bootstrap internal validation was not performed because the limited number of events would produce unstable optimism estimates; this is acknowledged as a limitation. All calibration and discrimination estimates should be interpreted as apparent/in-sample performance.

**Table 3:** Independent predictive factors for identifying ectopic pregnancy using multiple logistic regression analysis.

Variable	β	SE	Wald χ^2^	OR (95% CI)	*P* value
Prior ectopic pregnancy	1.466	0.695	4.45	4.33 (1.11–16.91)	0.035
Log progesterone	−1.854	0.458	16.39	0.157 (0.064–0.385)	<0.001
Endometrial thickness	−0.113	0.091	1.54	0.893 (0.747–1.068)	0.215
Abnormal adnexal ultrasound	3.258	0.639	25.99	26.00 (7.43–90.99)	<0.001
M6NP high-risk	2.789	0.668	17.43	16.27 (4.39–60.30)	<0.001

β-hCG-related variables were excluded from the final model due to multicollinearity with the M6NP high-risk classification. The final combined model included the M6NP high-risk classification (≥5%) as a binary variable; the coefficients are shown in Table 3.

**Figure 4:**
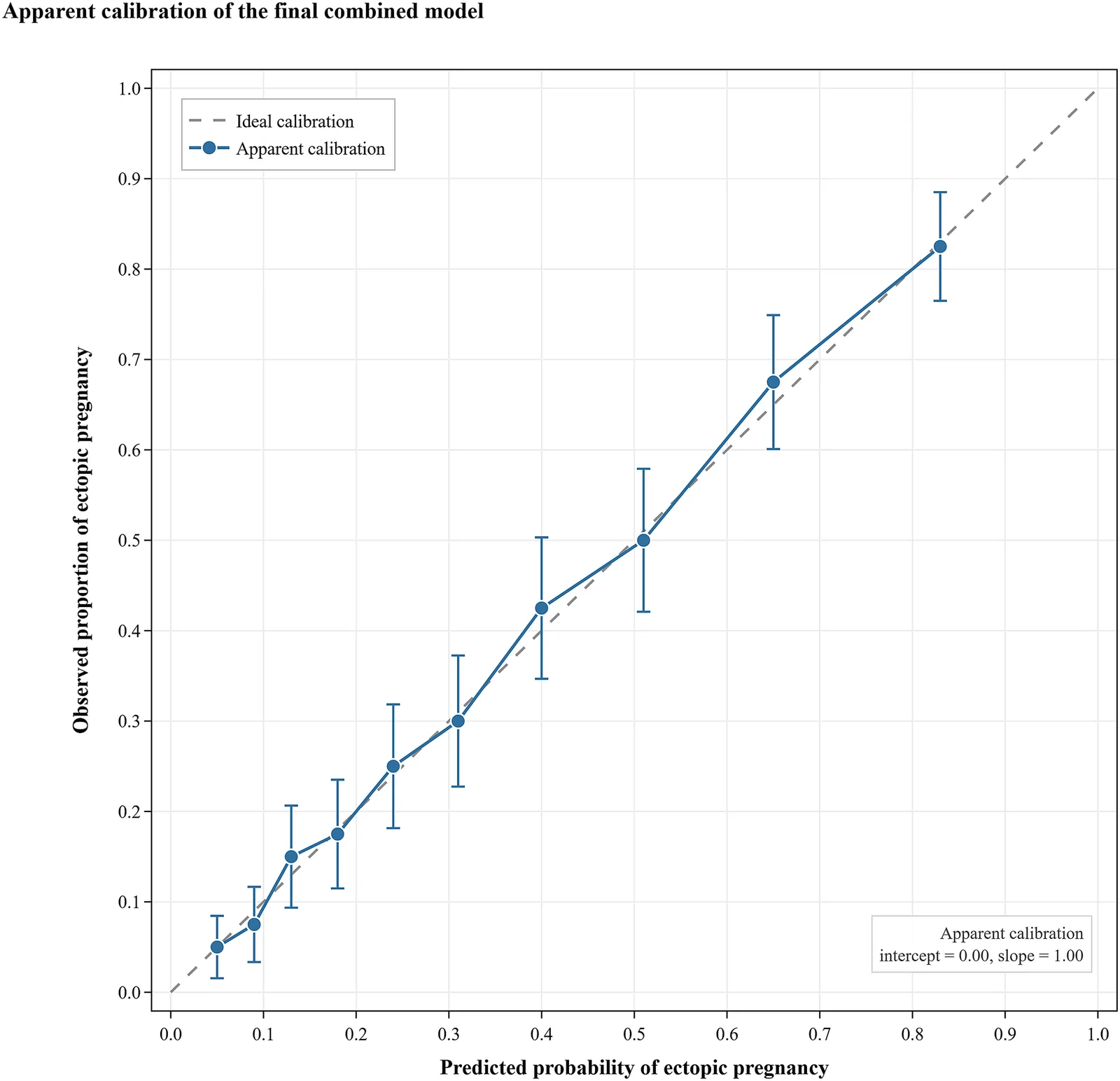
Apparent/in-sample calibration plot for the final combined logistic regression model. This is an apparent calibration plot; bootstrap internal validation was not performed, and calibration and discrimination estimates should be interpreted as apparent/in-sample performance.

### 3.4 ROC curve analysis of various indicators for diagnosing ectopic pregnancy

Table 4 ROC curve analysis shows that serum progesterone, endometrial thickness, abnormal ultrasound signs in the adnexa region, and the continuous M6NP predicted probability had AUC values ranging from 0.818 to 0.858 for the diagnosis of ectopic pregnancy, all of which had good diagnostic efficacy. After combining ultrasound with M6NP, the apparent AUC increased to 0.913; after further adding serum progesterone, the apparent AUC increased to 0.947, with improved sensitivity and specificity compared with a single indicator. Among them, the ultrasound + M6NP + progesterone combination showed the highest Youden index (0.828), but these values represent apparent performance from the same dataset used for model development and therefore require external validation before clinical application. For each indicator, the exact TP, FP, TN, and FN counts are now provided in Table 4, and all sensitivity, specificity, PPV, and NPV 95% CIs were recalculated from these integer counts using one consistently specified Wilson-score implementation. The recalculated intervals replace all previously reported intervals. See Table 4.

**Table 4:** ROC curve analysis of single and combined indicators for diagnosing ectopic pregnancy.

Indicator	AUC (95% CI)	Cut-off	TP	FP	TN	FN	Sensitivity % (95% CI)	Specificity % (95% CI)	PPV % (95% CI)	NPV % (95% CI)	Youden index
Serum progesterone	0.851 (0.792–0.910)	12.36 nmol/L	43	28	71	6	87.76 (75.8–94.3)	71.72 (62.2–79.6)	60.56 (48.9–71.1)	92.21 (84.0–96.4)	0.595
Endometrial thickness	0.818 (0.754–0.882)	9.75 mm	38	20	79	11	77.55 (64.1–87.0)	79.80 (70.9–86.5)	65.52 (52.7–76.4)	87.78 (79.4–93.0)	0.574
Abnormal adnexal ultrasound	0.852 (0.793–0.911)	—	39	9	90	10	79.59 (66.4–88.5)	90.91 (83.6–95.1)	81.25 (68.1–89.8)	90.00 (82.6–94.5)	0.705
M6NP predicted probability (continuous)	0.858 (0.800–0.916)	≥5%	41	12	87	8	83.67 (71.0–91.5)	87.88 (80.0–92.9)	77.36 (64.5–86.6)	91.58 (84.3–95.7)	0.716
Ultrasound + M6NP	0.913 (0.864–0.962)	0.28	43	13	86	6	87.76 (75.8–94.3)	86.87 (78.8–92.2)	76.79 (64.2–85.9)	93.48 (86.5–97.0)	0.746
Ultrasound + M6NP + progesterone	0.947 (0.908–0.986)	0.42	45	9	90	4	91.84 (80.8–96.8)	90.91 (83.6–95.1)	83.33 (71.3–91.0)	95.74 (89.6–98.3)	0.828

TP, FP, TN, and FN denote true-positive, false-positive, true-negative, and false-negative counts, respectively, based on the final pregnancy outcome as the reference standard. For all diagnostic indicators in Table 4, 95% confidence intervals for sensitivity, specificity, PPV, and NPV were recalculated using one consistently specified standard two-sided Wilson score method (z = 1.96) directly from the exact integer TP, FP, TN, and FN counts shown in the table. All previously reported intervals in this table have been replaced by these recalculated values. Because these analyses were exploratory, based on a limited number of events, and internally unvalidated, all intervals and performance estimates should be interpreted with caution.

### 3.5 Incremental diagnostic value of serum progesterone

Table 5 shows that after introducing serum progesterone into the ultrasound combined M6NP model, the apparent AUC of the model increased from 0.913 to 0.947, with a *Δ*AUC of 0.034. The DeLong test showed a statistically significant difference in AUC between the two models (*P* = 0.035). The integrated discrimination improvement (IDI) was 0.059 (95% CI 0.018–0.100), with a statistically significant difference (*P* = 0.005), indicating that the addition of serum progesterone provided a statistically significant improvement in discrimination in this development dataset. See Table 5 and Figure 5. Figure 5 has been updated to show AUCs of 0.913 and 0.947, *Δ*AUC = 0.034, and DeLong *P* = 0.035, consistent with Table 5.

**Table 5:** Analysis of diagnostic value of serum progesterone increment.

Model	AUC	*Δ*AUC	DeLong Z	*P* value	IDI (95% CI)	*P* value
Model A (ultrasound + M6NP)	0.913	Ref	—	—	—	—
Model B (Model A + progesterone)	0.947	0.034	2.112	0.035	0.059 (0.018–0.100)	0.005

**Figure 5:**
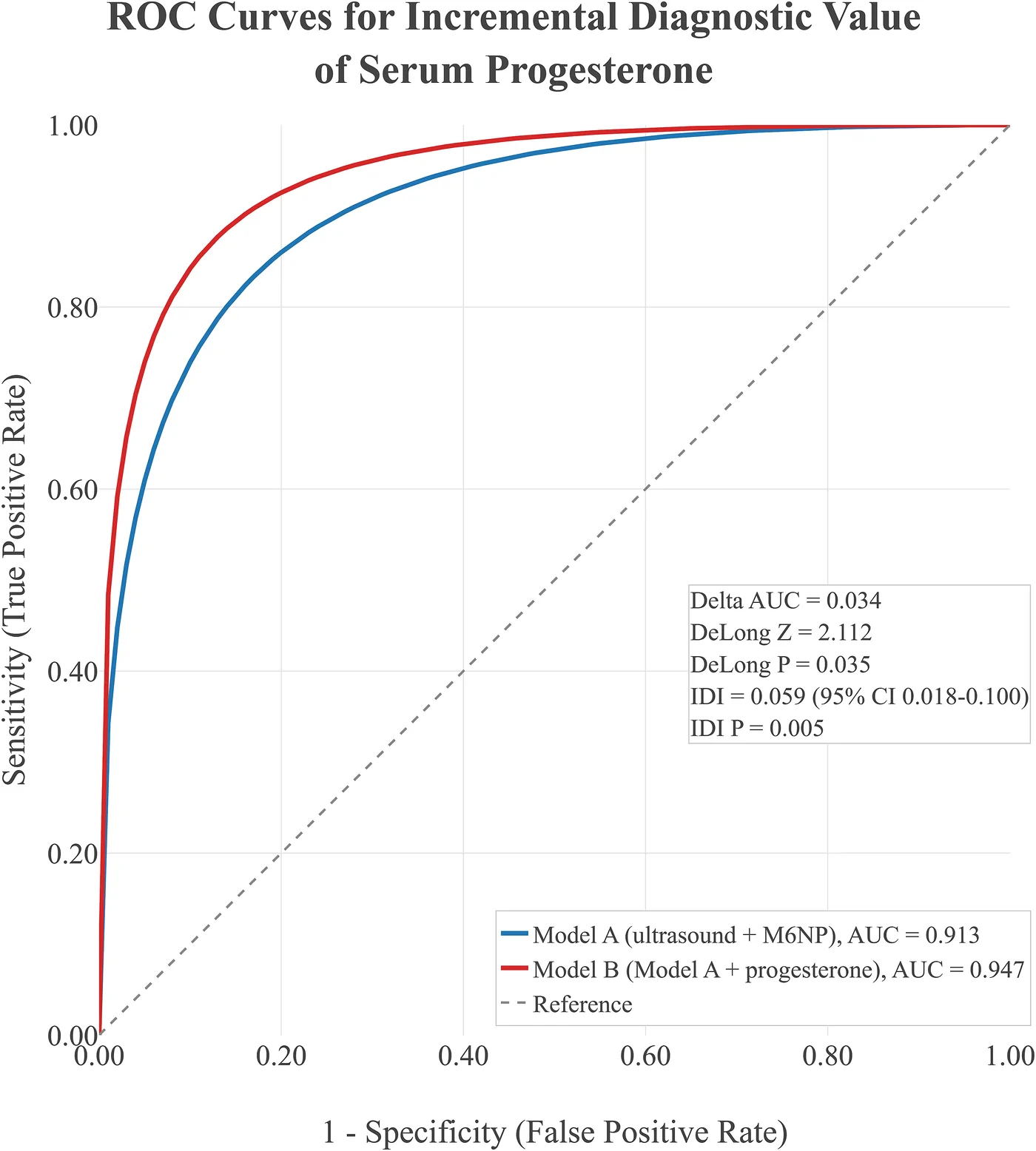
Incremental diagnostic value of serum progesterone: ROC curves for model A (ultrasound + M6NP) and model B (model A + progesterone).

### 3.6 Subgroup analysis: performance of progesterone diagnostic efficacy in different clinical features

As shown in Table 6, the AUC of serum progesterone remained at a relatively high level in subgroups stratified by age, duration of amenorrhea, and history of ectopic pregnancy; there was no statistically significant difference in AUC of the same indicator among the subgroups (*P* values for interaction were all>0.05). However, because the subgroup sample sizes were small, these non-significant interaction tests do not establish equivalence or “population stability/generalization ability.” The subgroup analyses should be regarded as exploratory. We did not report 95% confidence intervals for subgroup-specific AUCs because the limited number of events in each subgroup makes such intervals unstable and potentially misleading. In particular, the reported AUC of 0.899 in the 20-patient previous ectopic pregnancy subgroup should not be interpreted as evidence of stable diagnostic performance. See Table 6.

**Table 6:** Subgroup analysis of serum progesterone for diagnosing ectopic pregnancy.

Subgroup	n	AUC	Cutoff (nmol/L)	Sensitivity (%)	Specificity (%)	*P* for interaction
Age <35 years	102	0.842	12.41	86.36	70.37	0.524
Age ≥35 years	46	0.859	12.18	87.5	72	–
Days of amenorrhea <42 days	73	0.835	12.59	84.21	70.59	0.638
Days of amenorrhea ≥42 days	75	0.862	12.06	90.32	72.73	–
No prior ectopic pregnancy	128	0.840	12.28	86.49	72.53	0.432
Prior ectopic pregnancy present	20	0.899	12.73	90	75	–

### 3.7 Diagnosis of progesterone and ultrasound in different M6NP risk stratification

Table 7 shows that after stratification by M6NP risk, the AUC of progesterone combined with ultrasound in both low-risk and high-risk groups was higher than that of progesterone alone or ultrasound, and the Youden index for combined diagnosis was the highest, with improved sensitivity and specificity. The AUC of each individual and combined indicator in the high-risk stratification is higher than that in the low-risk stratification, indicating that the M6NP risk status may have a significant impact on the overall discriminative ability of the diagnostic model; However, the statistical test for direct comparison between groups is not presented in the table. See Table 7.

**Table 7:** Comparison of diagnostic efficacy by M6NP risk stratification.

Stratification	Indicator	AUC	Sensitivity (%)	Specificity (%)	Youden index
M6NP low-risk (*n*=95)	Serum progesterone	0.728	62.5	91.82	0.543
	Abnormal adnexal ultrasound	0.751	50	97.7	0.477
	Progesterone + ultrasound	0.854	75	90.8	0.658
M6NP high-risk (*n*=53)	Serum progesterone	0.874	90.24	72.73	0.63
	Abnormal adnexal ultrasound	0.886	85.37	91.67	0.77
	Progesterone + ultrasound	0.945	95.12	91.67	0.868

## 4 Discussion

Early identification of pregnancy location is still a core challenge in clinical practice of obstetrics and gynecology, and the use of serum progesterone and transvaginal ultrasound as traditional methods often have limitations when applied alone. From the results of this study, the median serum progesterone level in patients with ectopic pregnancy (EP) was only 10.80 nmol/L, significantly lower than the 48.50 nmol/L in the non EP group, and this difference reached statistical significance. The reason may be that during EP, the dysfunction of trophoblastic cells leads to insufficient stimulation of the corpus luteum, resulting in a corresponding decrease in progesterone secretion.

Previous studies have shown that a single progesterone measurement can help identify high-risk populations, and the cutoff values are mostly in the range of 10–15 nmol/L. The 12.36 nmol/L obtained in this study is basically consistent with this. However, it should be noted that due to individual differences in ovulation and luteal function affecting progesterone levels, its diagnostic threshold in specific populations still needs further calibration, which cannot be ignored (15).

Regarding the ultrasound features in the adnexal region, the abnormal detection rate of the EP group in this study was 79.59%, significantly higher than the 9.09% of the non EP group, and its AUC for individual diagnosis reached 0.852. These ultrasound features, such as tubal ring sign and mixed masses in the adnexal region, intuitively reflect the anatomical location and local blood supply changes of ectopic implantation. From a mechanistic perspective, the growth of the gestational sac in the fallopian tubes and other areas during ectopic pregnancy can induce local inflammatory reactions and edema, resulting in typical sonographic manifestations. However, the sensitivity range of diagnosing EP with adnexal masses in the literature is relatively large (60% -85%), and 79.59% of the patients in this study are at a moderate to high level. This may be related to the fact that all enrolled patients were in early pregnancy with unknown location and the lesions had not yet fully formed typical masses (16, 17). Difficult or atypical localizations, including ovarian, interstitial, cornual, and abdominal pregnancies, can be sonographically occult and may remain indeterminate even after structured preoperative assessment. After combining ultrasound with M6NP, the AUC increased to 0.913, and after further adding progesterone, the AUC increased to 0.947, significantly better than any single indicator, indicating that these parameters have complementary value (18).

Furthermore, this study included multiple factor analysis of initial *β* - hCG and 48 h dynamic changes, which showed that the mean change rate of *β*-hCG in the EP group was 15.20%, much lower than the 58.30% in the non-EP group (19). Notably, the large standard deviation (22.50%) implies that a considerable proportion of EP patients actually exhibited a decline in *β*-hCG levels over 48 h, consistent with the impaired and often regressive trophoblastic function in ectopic implantation (20).

Previous work has shown that continuous monitoring of *β* - hCG is an important means of distinguishing pregnancy outcomes, but there is still a risk of false positives when used alone. In this study, the OR value of dynamic changes in *β* - hCG was only 0.963 (95% CI: 0.948–0.977) (21). Although it was statistically significant, its clinical effect was relatively small, indicating its limited discriminatory ability as a single indicator (22).

The M6NP model (excluding initial progesterone) performed well in this analysis. The proportion of EP in the high-risk group is as high as 83.67%, with an AUC of 0.858. This model is an hCG-based model derived from the M6 framework and uses only log-transformed initial *β*-hCG concentration and the 48-hour hCG ratio, and its risk stratification ability has been confirmed by multiple external validation studies (9, 23).

However, when progesterone was added to the ultrasound + M6NP diagnostic strategy at the 48-hour reassessment time point, the AUC significantly increased from 0.913 to 0.947, and the integrated discrimination improvement (IDI) was 0.059 (*P* = 0.005). This incremental value indicates that progesterone provides unique information beyond the dynamic and ultrasound features of *β* - hCG - it is not just a marker of luteal function, but more likely to reflect early endocrine activity of the placenta, and this association deserves further exploration (24).

Regarding subgroup analysis, the diagnostic efficacy of progesterone in this study was not significantly modified by age, duration of amenorrhea, or previous history of ectopic pregnancy (interaction *P*-values were all>0.05). This result is consistent with some literature reports, indicating that progesterone has good stability as a biomarker. However, it should be pointed out that the sample size for subgroup analysis is relatively small. For patients with a history of ectopic pregnancy (only 20 cases), although the AUC is as high as 0.899, the confidence interval is wide (0.805–1.000), and the extrapolation of the results should be treated with caution (25).

In the high-risk stratification of M6NP, the AUC of progesterone combined with ultrasound reached 0.945, with a sensitivity of 95.12% and a specificity of 91.67%, demonstrating excellent comprehensive performance. These findings suggest that supplementing progesterone testing after initial risk stratification may have the potential to reduce unnecessary diagnostic surgeries or excessive follow-up, but this study did not directly evaluate these clinical outcomes and therefore no definitive conclusion can be drawn. On the contrary, in the low-risk group, even if the diagnostic role of progesterone is weakened, its high specificity (91.82%) still helps to exclude EP. This stratified diagnostic strategy may optimize clinical pathways and reduce medical resource consumption (26).

There are still several limitations to this study. Firstly, as a single center retrospective analysis, the sample size is limited (148 cases in total), and the EP group only had 49 cases, which may affect the statistical power and accuracy of subgroup analysis. In addition, inclusion required complete initial testing, 48-hour *β*-hCG measurements, adequate ultrasound imaging, complete follow-up, and ascertainable pregnancy outcome. Women requiring immediate treatment or lacking complete 48-hour data may have been systematically excluded, potentially introducing selection bias and inflating the apparent diagnostic performance. Secondly, despite the use of the M6NP model and clear gold standards, some patients with unknown pregnancy locations ultimately have classification bias in determining outcomes through clinical follow-up. Thirdly, although we have completely and explicitly defined the prediction time point as the 48-hour reassessment visit and have specified that abnormal adnexal ultrasound findings are those obtained at that follow-up scan, the possibility of temporal information leakage cannot be entirely excluded, as the precise timing of the follow-up ultrasound relative to the 48-hour blood draw was not uniformly recorded; this should be addressed in future prospective studies with standardized timing of all assessments. The intended clinical application should also be validated prospectively, because the present model is designed for risk re-stratification and follow-up planning at the 48-hour reassessment, not for immediate diagnosis or treatment decisions at the initial visit. In addition, unknown confounding factors such as a history of luteal insufficiency and recent drug use were not included in the analysis, and the results require further validation through prospective multicenter studies. Finally, because bootstrap internal validation was not performed, all calibration and discrimination estimates presented in this study should be considered apparent/in-sample performance and the absence of internal validation remains a key limitation.

Overall, this study suggests that serum progesterone combined with transvaginal ultrasound may have incremental diagnostic value in distinguishing early ectopic pregnancy, especially after preliminary stratification in the M6NP model. However, because the study included only 49 EP cases and was not prospectively powered for multivariable modelling or incremental AUC testing, all multivariable performance estimates and subgroup analyses must be regarded as exploratory and hypothesis-generating. These findings provide preliminary evidence that the combined strategy may improve diagnostic accuracy, but prospective external validation in larger cohorts is required before the strategy can be considered a reproducible clinical decision-support tool. Future research should focus on establishing a multi parameter prediction model that includes progesterone and validating its generalizability in larger real-world cohorts.

## Data Availability

The original contributions presented in the study are included in the article/supplementary material, further inquiries can be directed to the corresponding author.

## References

[1] Istrate-Ofiţeru A-M Ruican D Niculescu M Nagy RD Roşu G-C Petrescu A-M. Ovarian ectopic pregnancy: the role of complex morphopathological assay. Review and case presentation. Rom J Morphol Embryol. (2020) 61(4):985–97. 10.47162/RJME.61.4.01 PMC8343605 34171048

[2] Pereira PP Cabar FR Gomez ÚT Francisco RPV. Pregnancy of unknown location. Clinics. (2019) 74:(e1111). 10.6061/clinics/2019/e1111 PMC6784610 31618321

[3] Hendriks E Rosenberg R Prine L. Ectopic pregnancy: diagnosis and management. Am Fam Physician. (2020) 101(10):599–606. 32412215

[4] Beer LA Yin X Ding J Senapati S Sammel MD Barnhart KT. Identification and verification of plasma protein biomarkers that accurately identify an ectopic pregnancy. Clin Proteomics. (2023) 20(1):37. 10.1186/s12014-023-09425-w 37715129 PMC10503165

[5] Sundermeyer RL LeFevre N Kolker A Williamson B. Common issues in prenatal care: first trimester bleeding. FP Essent. (2023) 525:7–12. 36780553

[6] Kirk E Bottomley C Bourne T. Diagnosing ectopic pregnancy and current concepts in the management of pregnancy of unknown location. Hum Reprod Update. (2014) 20(2):250–61. 10.1093/humupd/dmt047 24101604

[7] Reza C Hammerum MS Michaud SJ Klarskov N Amirian I. Evaluating the performance of the M6 model without initial serum progesterone (M6NP) and the NICE guidelines in a Danish pregnancy of unknown location (PUL) population. Eur J Obstet Gynecol Reprod Biol. (2025) 312:114526. 10.1016/j.ejogrb.2025.114526 40517510

[8] Van Calster B Bobdiwala S Guha S Van Hoorde K Al-Memar M Harvey R. Managing pregnancy of unknown location based on initial serum progesterone and serial serum hCG levels: development and validation of a two-step triage protocol. Ultrasound Obstet Gynecol. (2016) 48(5):642–9. 10.1002/uog.15864 26776599

[9] Kyriacou C Ledger A Bobdiwala S Ayim F Kirk E Abughazza O. Updating M6 pregnancy of unknown location risk-prediction model including evaluation of clinical factors. Ultrasound Obstet Gynecol. (2024) 63(3):408–18. 10.1002/uog.27515 37842861

[10] Bobdiwala S Saso S Verbakel JY Al-Memar M Van Calster B Timmerman D. Diagnostic protocols for the management of pregnancy of unknown location: a systematic review and meta-analysis. BJOG. (2019) 126(2):190–8. 10.1111/1471-0528.15442. PMID: 30129999. 30129999

[11] Kabzińska-Turek M Balajewicz-Nowak M Piskorz T Hosiawa V. Pregnancy of unknown location - still diagnostics and therapeutic problem. Folia Med Cracov. (2025) 65(1):157–68. 10.24425/fmc.2025.156118 41061097

[12] Memtsa M Jauniaux E Gulbis B Jurkovic D. Evaluation of maternal serum biomarkers in predicting outcome of successful expectant management of tubal ectopic pregnancies. Eur J Obstet Gynecol Reprod Biol. (2020) 250:61–5. 10.1016/j.ejogrb.2020.04.009 32387894

[13] Ozel S Alkan M Tokmak A Oksuzoglu A Kaya M Aktulay A. Relationship between polycystic ovarian morphology and ectopic pregnancy. J Reprod Infertil. (2021) 22(1):32–7. 10.18502/jri.v22i1.4993 33680883 PMC7903666

[14] Reid S Casikar I Barnhart K Condous G. Serum biomarkers for ectopic pregnancy diagnosis. Expert Opin Med Diagn. (2012) 6(2):153–65. 10.1517/17530059.2012.664130 23480657

[15] Han X Qian X Wan X Chen Y Chen L. Differential diagnosis of non-molar gestational trophoblastic neoplasia with ectopic pregnancy by clinical-pathological features. Arch Gynecol Obstet. (2024) 310(4):2161–6. 10.1007/s00404-024-07702-z. PMID: 39210069. 39210069

[16] Verhaegen J Gallos ID Van Mello NM Abdel-Aziz M Takwoingi Y Harb H. Accuracy of single progesterone test to predict early pregnancy outcome in women with pain or bleeding: meta-analysis of cohort studies. Br Med J. (2012) 345:e6077. 10.1136/bmj.e6077 PMC3460254 23045257

[17] Friis Petersen J Grynnerup AG-A Mitchell NH Løssl K Sørensen S Lindhard A. Soluble urokinase plasminogen activator receptor (suPAR) as a biomarker of early pregnancy location and viability compared with hCG, progesterone and estradiol. J Reprod Immunol. (2020) 138:103103. 10.1016/j.jri.2020.103103 32145561

[18] Casikar I Reid S Condous G. Ectopic pregnancy: ultrasound diagnosis in modern management. Clin Obstet Gynecol. (2012) 55(2):402–9. 10.1097/GRF.0b013e31825109bd 22510621

[19] Kirk E Papageorghiou AT Condous G Tan L Bora S Bourne T. The diagnostic effectiveness of an initial transvaginal scan in detecting ectopic pregnancy. Hum Reprod. (2007) 22(11):2824–8. 10.1093/humrep/dem283 17855406

[20] Bobdiwala S Al-Memar M Farren J Bourne T. Factors to consider in pregnancy of unknown location. Womens Health (Lond. (2017) 13(2):27–33. 10.1177/1745505717709677 28660799 PMC5557179

[21] Barnhart KT Sammel MD Rinaudo PF Zhou L Hummel AC Guo W. Symptomatic patients with an early viable intrauterine pregnancy: hCG curves redefined. Obstet Gynecol. (2004) 104(1):50–5. 10.1097/01.AOG.0000128174.48843.12. PMID: 15229000. 15229000

[22] van Mello NM Mol F Opmeer BC Ankum WM Barnhart K Coomarasamy A. Diagnostic value of serum hCG on the outcome of pregnancy of unknown location: a systematic review and meta-analysis. Hum Reprod Update. (2012) 18(6):603–17. 10.1093/humupd/dms035 22956411

[23] Fistouris J Bergh C Strandell A. Pregnancy of unknown location: external validation of the hCG-based M6NP and M4 prediction models in an emergency gynaecology unit. BMJ Open. (2022) 12(11):e058454. 10.1136/bmjopen-2021-058454. PMID: 36446455; PMCID: PMC9716941. 36446455 PMC9716941

[24] Papageorgiou D Sapantzoglou I Prokopakis I Zachariou E. Tubal ectopic pregnancy: from diagnosis to treatment. Biomedicines. (2025) 13(6):1465. 10.3390/biomedicines13061465 40564186 PMC12191334

[25] Maheut C Panjo H Capmas P. Diagnostic accuracy validation study of the M6 model without initial serum progesterone (M6(NP)) in triage of pregnancy of unknown location. Eur J Obstet Gynecol Reprod Biol. (2024) 296:360–5. 10.1016/j.ejogrb.2024.03.010 38552504

[26] Reza C Amirian I Hammerum MS Klarskov N Husby K. Activity-based costing in the management of pregnancy of unknown location: comparing current practice in Denmark with the M6NP model, and the NICE guidelines. Eur J Obstet Gynecol Reprod Biol. (2026) 321:115086. 10.1016/j.ejogrb.2026.115086. PMID: 41887127. 41887127

